# *QuickStats*: Percentage of Adults Aged ≥65 Years Who Had Ever Received Pneumococcal Vaccination,* by Age Group — National Health Interview Survey,^†^ United States, 2000–2018

**DOI:** 10.15585/mmwr.mm6932a8

**Published:** 2020-08-14

**Figure:**
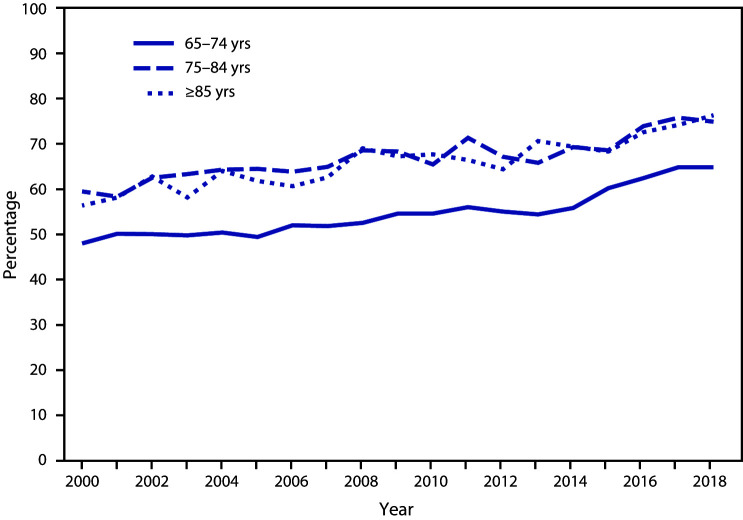
During 2000–2018, the percentage of adults aged ≥65 years who had ever received a pneumonia vaccine increased. The percentage increased from 48.0% to 64.8% among adults aged 65–74 years, from 59.5% to 74.9% among adults aged 75–84 years, and from 56.4% to 76.3% among adults aged ≥85 years. Throughout the period, adults aged 65–74 years were less likely to have ever received a pneumonia vaccine than adults aged ≥75 years.

**Source:** National Health Interview Survey, 2000–2018. https://www.cdc.gov/nchs/nhis.htm.

For more information on this topic, CDC recommends the following link: https://www.cdc.gov/vaccines/hcp/acip-recs/vacc-specific/pneumo.html.

